# Beyond abuse and neglect: validation of the childhood interpersonal trauma inventory in a community sample of adults

**DOI:** 10.3389/fpsyt.2024.1358475

**Published:** 2024-02-29

**Authors:** Maxime Legendre, Tristan Milot, Michel Rousseau, Roxanne Lemieux, Julia Garon-Bissonnette, Nicolas Berthelot

**Affiliations:** ^1^ Department of Nursing Sciences, Université du Québec à Trois-Rivières, Trois-Rivières, QC, Canada; ^2^ Centre d’études Interdisciplinaires sur le Développement de l’enfant et la Famille (CEIDEF), Trois-Rivières, QC, Canada; ^3^ CERVO Brain Research Center, Université Laval, Québec, QC, Canada; ^4^ Interdisciplinary Research Center on Intimate Partner Relationship Problems and Sexual Abuse (CRIPCAS), Université de Montréal, Montréal, QC, Canada; ^5^ Groupe de Recherche et d’Intervention auprès des Enfants Vulnérables et Négligés (GRIN), Université du Québec à Trois-Rivières, Trois-Rivières, QC, Canada; ^6^ Department of Psychoeducation, Université du Québec à Trois-Rivières, Trois-Rivières, QC, Canada; ^7^ Centre de Recherche Universitaire sur les Jeunes et les Familles (CRUJeF), CIUSSS de la Capitale-Nationale, Québec, QC, Canada; ^8^ Peabody College of Education and Human Development, Vanderbilt University, Nashville, TN, United States

**Keywords:** adverse childhood experience, childhood trauma, maltreatment, PTSD, questionnaire

## Abstract

**Introduction:**

Childhood trauma is not restricted to abuse or neglect and other potentially traumatic experiences need to be pondered in practice and research. The study aimed to collect validity evidence of a new measure of exposure to a broad range of potentially traumatic experiences, the Childhood Interpersonal Trauma Inventory (CITI), by evaluating whether the CITI provides important additional information compared to a gold standard measure of childhood trauma.

**Methods:**

The sample consisted of 2,518 adults who completed the CITI and self-reported measures of trauma (Childhood Trauma Questionnaire; CTQ) and psychiatric symptoms (PTSD Checklist for DSM-5; Kessler Psychological Distress Scale; Dissociative Experiences Scale).

**Results:**

First, the sensitivity to properly detect participants having been exposed to childhood maltreatment, as measured by the CTQ (here used as the gold standard), ranged between 64.81% and 88.71%, and the specificity ranged between 68.55% and 89.54%. Second, hierarchical regressions showed that the CITI predicted between 5.6 and 14.0% of the variance in psychiatric symptoms while the CTQ only captured a very small additional part of variance (0.3 to 0.7%). Finally, 25% (n = 407) of CTQ-negative participants screened positive at the CITI. The latter reported higher severity of psychiatric symptoms than participants without trauma, suggesting that the CITI permits the identification of adults exposed to significant traumas that remain undetected using other well-validated measures.

**Discussion:**

The findings underscore the utility of the CITI for research purposes and the latter’s equivalence to a gold standard self-reported questionnaire to predict negative outcomes.

## 1 Introduction

Childhood trauma is frequent in community samples of adults (1, 2) and has been associated with deleterious lifelong consequences including psychiatric disorders (3, 4) and poor functioning (5–7). A personal history of childhood trauma in adult parents has also been associated with altered parenting (8) and poor outcomes in offspring, including higher rates of exposure to maltreatment (9, 10). Most importantly, patients exposed to childhood trauma would present distinct psychiatric disorders at the molecular, physiological and phenotypical levels than not-exposed patients suffering from the same disorders (11–13). The former would indeed show worsened psychiatric conditions (14, 15), poorer evolution of illness and treatment (16), earlier onset of illness (17), and may require specific treatments (18). This has recently led Teicher, Gordon and Nemeroff (19) to formulate a set of recommendations to leverage science and practice, including investigating for childhood trauma in clinical practice when assessing or treating patients with mental health issues, introducing a maltreatment-related subtype into diagnostic nosology, and considering childhood trauma as a key variable in clinical trials and basic research on the biological basis of psychiatric disorders. Accordingly, while it is important to recognize that screening for childhood trauma in primary care settings could be ineffective and even detrimental (20, 21) when it is not implemented cautiously using a trauma-informed framework (22), the science is clear that childhood trauma cannot be overlooked and needs to be considered by clinicians and researchers in order to adjust interventions and accelerate discoveries (19, 23–36).

Recent findings highlighted that childhood trauma is not restricted to abuse or neglect and that other potentially traumatic experiences need to be pondered in practice and research given their long-lasting repercussions on health and functioning. For instance, peer and sibling bullying, a particular form of childhood trauma relatively common in the general population (27, 28), has been associated with a wide range of short and long term negative outcomes including social isolation (29), internalized and externalized problems (30, 31), post-traumatic stress disorder (32), and many other psychopathologies such as anxiodepressive disorders (33). Parentification or role reversal has been associated with persisting negative effects on social-emotional development (34) and internalized and externalized behaviors (35), and seems to mimic some effects of parental neglect. Similar findings were found for other types of interpersonal traumas such as witnessing intimate partner violence (36), living with a parent having a substance-use disorder (37) or who attempt suicide (38), parental alienation (39, 40) and parental overprotection-overcontrol (41). Indeed, these experiences share many similarities with abuse or neglect in terms of their age of onset, chronicity, repercussions, and most importantly the fact that they involve the failure of significant others to provide children with a safe environment and to respond adequately to their needs.

Despite the importance of taking these experiences into account and the absence of empirical evidence suggesting that they represent minor forms of trauma especially when they persist over time or accumulate, most existing instruments assessing childhood trauma do not consider such potentially traumatic experiences. For instance, the most frequently used self-report measure of childhood trauma, the Childhood Trauma Questionnaire (CTQ; 42), covers the typical forms of childhood maltreatment (i.e., physical, sexual, and emotional abuse, as well as physical and emotional neglect) but does not capture any other types of interpersonal traumas such as bullying, domestic violence, and household dysfunction. The Adverse Childhood Experience (ACE; 43), another widely used questionnaire, covers traumatic experiences not included in the CTQ such as parental domestic violence, incarceration and divorce or separation. However, this questionnaire has been criticized because it encompasses few traumatic experiences and the wording of the items is very broad and vague making it difficult to interpret results (44).

Overall, although the CTQ and the ACE are widely used, they only cover a limited number of potentially traumatic experiences. This raises the possibility of false-negatives in that some participants with low to moderate scores on these instruments may have experienced many other types of interpersonal traumas not covered by these questionnaires. In clinical practice, this may eventually lead to some patients not receiving adapted treatments or not being involved in available trauma-centered or trauma-informed interventions when they would clearly benefit from them. We believe there is a need for an easy-to-use screening tool that could be used in various clinical and research settings to assess a broader range of potentially traumatic experiences. To be effective, such a screening tool should be (1) easy to administer and interpret (2), widely available, and (3) empirically supported (45). In the case of childhood trauma, this means, for instance, being as concise as possible but broad enough to cover a large spectrum of potentially traumatic experiences that were shown to be associated with poor mental health.

Accordingly, in the current study, we aimed to collect validity evidence of the Childhood Interpersonal Trauma Inventory (CITI; Lemieux R and Berthelot)^1^, a screening tool that (a) can be administered and interpreted simply and rapidly in clinical practice as well as in research, (b) encompasses a large range of potentially traumatic experiences, some remaining undetected by current measures of interpersonal traumas, (c) offers a global severity index as well as precise information on the types of traumatic experiences, and (d) is equivalent to the most widely used instruments in the prediction of negative outcomes. More precisely, the study aimed to evaluate (1) the sensitivity and specificity of the CITI to detect abuse and neglect, using the CTQ as the gold standard (2), the convergent validity between the CITI and the CTQ and incremental validity of the CITI, and (3) the relevance and utility of the CITI by showing that some participants classified as “non-exposed” when using the CTQ had experienced significant interpersonal traumas that are severe enough to show associations with negative outcomes.

## 2 Materials and methods

### 2.1 Participants

Data for this study were collected in the course of a longitudinal research aiming to evaluate the impact of childhood trauma on parenthood and the effect of a trauma-centered intervention offered to pregnant women who experienced childhood adversity. Participants were recruited through presentation of the research at pregnancy-related medical appointments and through advertisements on social media. The sample comprises 2518 adults expecting a child, mainly pregnant women (93%), with a mean age of 29.6 (SD = 4.5, range = 18-55). To be included in the study, participants had to be 18 years old or older, have sufficient reading skills to complete self-reported instruments and awaiting a child. There were no exclusion criteria based on psychiatric diagnoses. Sociodemographic characteristics of the sample are presented in **Table 1**.

**Table 1:** Description of the sample.

**Age**, mean (SD)	29.59 (4.47)
Sex, n (%)	
Women	2324 (92.52%)
Men	182 (7.24%)
Other	6 (0.24%)
Ethnicity, n (%)	
White	2376 (95.42%)
Other	114 (4.58%)
Education level, n (%)	
High school diploma or less	244 (9.72%)
Collegial or professional training	1057 (42.11%)
University degree	1209 (48.17%)
Occupation, n (%)	
Full-time worker	701 (59.51%)
Part-time worker	73 (6.20%)
Preventive withdrawal	241 (20.46%)
Other	163 (13.83%)
Annual household income, n (%)	
Can $64 999 or less	676 (27.04%)
Can $65 000 - 94 999$	784 (31.36%)
Can $95 000$ or more	1040 (41.60%)
Marital status, n (%)	
Married	429 (17.11%)
Common-law union	1968 (78.50%)
Single	110 (4.39%)
**CITI**, mean (SD)	4.48 (4.95)
**CTQ**, mean (SD)	34.51 (12.58)
**PCL-5**, mean (SD)	13.01 (12.34)
**K10**, mean (SD)	20.13 (6.37)
**DES**, mean (SD)	15.78 (16.67)

Total N for Sociodemographic data, 2490; CITI and CTQ, 2518; PCL-5, 2430; K10, 2490; DES, 1951. CITI, Childhood Interpersonal Trauma Inventory; CTQ, Childhood Trauma Questionnaire; PCL-5, Post-Traumatic Stress Disorder Checklist for DSM-5; K10, Kessler Psychological Distress Scale; DES, Dissociative Experiences Scale; SD, Standard Deviation.

### 2.2 Procedure

Participants were recruited at pregnancy-related medical appointments (n = 1171) between April 2018 and March 2021 in prenatal clinics in the province of Quebec, Canada. These participants were informed of the study by nurses or clinic staff and those who agreed to participate were subsequently contacted during the second trimester of pregnancy and invited to complete a set of online questionnaires. Participants recruited from social media platforms (n = 1347) were recruited online between April 2nd and April 13th 2020 during the first COVID-19 mandatory lockdown in the province of Quebec. This study received ethical approval from our University (CER-15-210-07; CER-16-226-10; CER-20-266-10) and Health Care Center (CER-2014-027; CER-2016-016-11) ethic committees. The order of the presentation of the two measures of trauma (CTQ and CITI) was different in the two samples to avoid the risk of a measure having a priming effect on responses to the following instrument. However, the order of presentation was identical for all participants within a given sample. ANOVAs controlling for age, education and income confirmed that there were no differences between samples on CITI scores (*p* > 0.05).

### 2.3 Measures

#### 2.3.1 Childhood interpersonal trauma inventory

The Childhood Interpersonal Trauma Inventory (CITI; Lemieux R and Berthelot)^1^ was initially developed in the course of a research project evaluating a trauma-centered intervention for adults, awaiting a child, who had experienced interpersonal trauma during their childhood (46). In the course of that study, we found ourselves excluding participants who appeared to have experienced significant adversity in attachment relationships which was not properly captured by the CTQ. As we were not aware of other brief instruments that could fulfill that purpose, we developed this checklist. A panel of three clinician-researchers with expertise in childhood trauma was formed. A serial process was used, during which a first member suggested a preliminary list of interpersonal traumas. A second member independently reviewed the items, made comments, and added other relevant potentially traumatic experiences, and so on until a consensus was reached among the three members. The CITI is a self-reported questionnaire originally developed in French that covers 33 potentially traumatic experiences that may occur before 18 years old (see **Supplementary Material**). For each potentially traumatic experience, respondents must indicate by “yes” or “no” whether they consider that this experience applies to something they experienced before the age of 18. At the end, respondents can also indicate potentially traumatic experiences that were not covered by the questionnaire. Whereas this last item offers interesting information, especially for clinicians, it is not included in the cumulative score. Similar to the CTQ and the ACE, the items cover each of the five types of childhood maltreatment (sexual abuse, physical abuse, emotional abuse, physical neglect and emotional neglect) as well as other potentially traumatic experiences. The CITI produces a cumulative score obtained by adding the scores (0 or 1) to the 33 items. For the current study, we also computed six dichotomous scores reflecting the presence/absence of the five types of childhood maltreatment assessed with the CTQ using a method defined in the **Supplementary Material**.

#### 2.3.2 Childhood trauma questionnaire

The French version (47) of the Childhood Trauma Questionnaire (CTQ; 42) was used for convergent validity. This self-reported measure covers five types of childhood maltreatment (sexual abuse, physical abuse, emotional abuse, physical neglect and emotional neglect) using 28 items rated on a 5-point Likert scale, ranging from 1 (never true) to 5 (very often true). Cut-offs are validated for each subscale (sexual abuse ≥ 8, physical abuse ≥8, emotional abuse ≥ 10, physical neglect ≥ 8, and emotional neglect ≥ 15; 48). Previous studies demonstrated the validity of the 28-item version of the CTQ across clinical and community samples (49, 50). The internal consistency in this study was α = .83 for the total score and between α = .76 and.94 for the five subscales.

#### 2.3.3 Post-traumatic stress disorder checklist for DSM-5

The French version (51) of the PTSD Checklist for DSM-5 (PCL-5; 52) was used for incremental validity. This self-reported measure covers post-traumatic stress (PTSD) symptoms with 20 items rated on a 5-point Likert scale ranging from 0 (not at all) to 4 (always). The clinical cut-off (≥ 33) would be highly predictive of a DSM-5 diagnosis of PTSD (53). Previous studies demonstrated that the French and English versions of PCL-5 have good reliability (internal consistency, temporal stability, test-retest) and convergent validity (51–53). The internal consistency in this study was α = .92.

#### 2.3.4 Kessler psychological distress scale

The French version (54) of the Kessler Psychological Distress Scale (K10; 55) was used for incremental validity. This self-reported measure covers anxiety and depression symptoms with 10 items rated on a 5-point Likert scale ranging from 1 (none of the time) to 5 (all of the time). The clinical cut-off was established at ≥30 as 76.3% of adults with scores reaching this cut-off would meet the diagnostic criteria for an anxiety, affective or substance use disorder (56). Previous studies demonstrated that the K10 is adequate for screening mood and anxiety disorders in pregnant women (57). The internal consistency in this study was α = .88.

#### 2.3.5 Dissociative experiences scale

The French version (58) of the Dissociative Experiences Scale (DES; 59) was used for incremental validity. This self-reported measure covers dissociative symptoms with 28 items rated on a 11-point Likert scale ranging from 0% to 100% (according to the extent to which each statement reflects the experience of the participant). The clinical cut-off (≥ 30) would be highly predictive of a dissociative disorder (60). Previous studies demonstrated that the DES has good construct validity and reliability (58–60). The internal consistency in this study was α = .88.

### 2.4 Data analysis

Data analyses were performed using the Statistical Package for Social Sciences (SPSS), version 24. Prior to analyses, all distributions were inspected, no outlier was identified, and it was determined that no transformation was needed. The percentage of missing data was 0% for the CITI, 1.75% for the CTQ, 1.11% for the PCL-5, 3.49% for the K10, 22.52% for the DES, and 1.11% for the sociodemographic data. Analyses were carried out according to the available data. The sensitivity and specificity of the CITI for detecting the five types of maltreatment assessed in other widely used instruments were calculated using the CTQ as the gold standard measure. Sensitivity represents the number of participants detected by the CITI among those detected by the CTQ, i.e., the proportion of True + properly detected. Specificity represents the number of participants excluded by the CITI among those excluded by the CTQ, i.e., the proportion of True - properly excluded. For each of the five types of maltreatment, endorsing one item of the CITI related to this type of maltreatment was sufficient to be considered “positive” (see the electronic supplement for more details). Pearson correlations were used to evaluate the convergent validity between the CTQ and the CITI. To evaluate incremental validity of the CITI, hierarchical linear regressions using the CITI and the CTQ as predictors of post-traumatic stress (PCL-5), anxiodepressive (K10), and dissociative symptoms (DES) were performed. In the regressions, the CITI total score was entered in Step 1, and the CTQ total score was entered in Step 2 to see if a significant part of variance in outcomes not explained by the CITI could be explained by the CTQ. To demonstrate the relevance and utility of the CITI, one-way ANOVAs were performed with the same three outcomes to compare three groups of participants: (1) Positive at the CTQ, (2) Negative at the CTQ and Positive at the CITI and, (3) Negative at both questionnaires. Respondents were considered to have experienced significant trauma on the CITI when they endorsed ≥ 4 items, a cut-off that has been associated with higher odds of psychological and physical consequences with similar screening instruments such as the ACE questionnaire (61, 62). A priori sample size estimation suggests that a sample of 1840 participants or more would permit small effects to be detected using one-way ANOVAs with three groups and that a sample of 2140 participants or more would permit a 1% variance change to be detected using linear regressions with two predictors.

## 3 Results

Overall, the sensitivity and specificity of the CITI to properly detect participants who experienced abuse or neglect according to the CTQ was of 79.40% and 75.39% respectively. The sensitivity to properly detect participants with the five types of maltreatment ranged between 64.81% and 88.71%, and the specificity ranged between 68.55% and 89.54% (**Table 2**). More precisely, sexual abuse, physical abuse, and emotional neglect with the CTQ were detected by the CITI 82% to 89% of the time whereas emotional abuse and physical neglect were respectively detected 75% and 65% of the time. As regards specificity, sexual abuse, physical abuse, and physical neglect were ruled out 87% to 90% of the time whereas emotional abuse and emotional neglect were respectively ruled out 82% and 69% of the time.

**Table 2:** Accuracy in percentage of the CITI using the CTQ as gold standard.

	True +	True -	Correct	False +	False -	Errors	Sensitivity	Specificity
**Total score**	27.25	49.52	76.77	16.16	7.07	23.23	79.40	75.39
**Sexual abuse**	9.44	77.54	86.98	11.03	1.99	13.02	82.58	87.54
**Physical abuse**	6.45	79.71	86.16	12.73	1.11	13.84	85.26	86.22
**Emotional abuse**	14.78	66.30	81.08	14.14	4.78	18.92	75.56	82.42
**Physical neglect**	10.62	74.87	85.49	8.75	5.76	14.51	64.81	89.54
**Emotional neglect**	10.96	60.08	71.04	27.57	1.39	28.96	88.71	68.55

CITI, Childhood Interpersonal Trauma Inventory; CTQ, Childhood Trauma Questionnaire. CTQ cut-offs are as follows: sexual abuse ≥ 8, physical abuse ≥8, emotional abuse ≥ 10, physical neglect ≥ 8, and emotional neglect ≥ 15.

As regards convergent and incremental validity, the CITI showed a significant correlation of 0.81 with the CTQ. Post-traumatic, anxiodepressive and dissociative symptoms were all significantly associated with the CITI scores, with values ranging from 0.24 to 0.37 (**Table 3**). A Fisher’s Z transformation of Pearson coefficient shown that the correlations between CTQ total score and symptom scores were not significantly different than the strength of the correlation between CITI total score and symptom scores (PCL-5: Z=0.80, p=0.21; K10: Z=1.48, p=0.07; DES: Z=0.37, p=0.36). Hierarchical regressions showed that the CITI explained 14.0% of the variance in post-traumatic stress symptoms, with an additional 0.7% part of variance explained by the CTQ. The second regression model showed that the CITI explained 6.2% of the variance in anxiodepressive symptoms, with no additional portion of variance explained by the CTQ. Finally, the third model showed that the CITI explained 5.6% of the variance in dissociative symptoms, with an additional 0.3% explained by the CTQ (see **Table 4**).

**Table 3:** Correlations between the CITI and the CTQ with the three outcomes.

	PCL-5	K10	DES
CITI			
Total score	.37*	.25*	.24*
Sexual abuse	.22*	.12*	.16*
Physical abuse	.20*	.12*	.14*
Emotional abuse	.27*	.19*	.16*
Physical neglect	.19*	.13*	.12*
Emotional neglect	.32*	.24*	.20*
Other potentially traumatic experiences	.35*	.23*	.22*
CTQ			
Total score	.35*	.21*	.23*
Sexual abuse	.21*	.12*	.15*
Physical abuse	.21*	.12*	.14*
Emotional abuse	.35*	.22*	.22*
Physical neglect	.25*	.14*	.17*
Emotional neglect	.30*	.21*	.18*

CITI, Childhood Interpersonal Trauma Inventory; CTQ, Childhood Trauma Questionnaire; PCL-5, Post-Traumatic Stress Disorder Checklist for DSM-5; K10, Kessler Psychological Distress Scale; DES, Dissociative Experiences Scale. *p < 0.001

**Table 4:** Hierarchical regression evaluating the contribution of scores at the CITI and the CTQ to PTSD, anxiodepressive and dissociative symptoms.

	*β*	*t*	*F*	*R*	*R* ^2^	Adj. *R* ^2^	*R* ^2^ change
PCL-5							
**Step 1**			389.85**	0.37	0.14	0.14	0.14**
CITI	0.37	19.75**					
**Step 2**			206.37**	0.38	0.15	0.15	< 0.01**
CITI	0.26	7.97**					
CTQ	0.14	4.45**					
K10							
**Step 1**			163.07**	0.25	0.06	0.06	0.06**
CITI	0.25	12.77**					
**Step 2**			82.04**	0.25	0.06	< 0.01	< 0.01
CITI	0.22	6.65**					
CTQ	0.03	1.00					
DES							
**Step 1**			115.71**	0.24	0.06	0.06	0.06**
CITI	0.24	10.76**					
**Step 2**			61.56**	0.24	0.06	0.06	< 0.01*
CITI	0.16	4.14**					
CTQ	0.10	2.65*					

CITI total score was entered in Step 1 and CTQ total score was entered in Step 2 to evaluate whether a significant part of variance in outcomes not explained by the CITI could be explained by the CTQ. CITI, Childhood Interpersonal Trauma Inventory; CTQ, Childhood Trauma Questionnaire; PCL-5, Post-Traumatic Stress Disorder Checklist for DSM-5; K10, Kessler Psychological Distress Scale; DES, Dissociative Experiences Scale. *p < 0.05 **p < 0.001.

**Figure 1:**
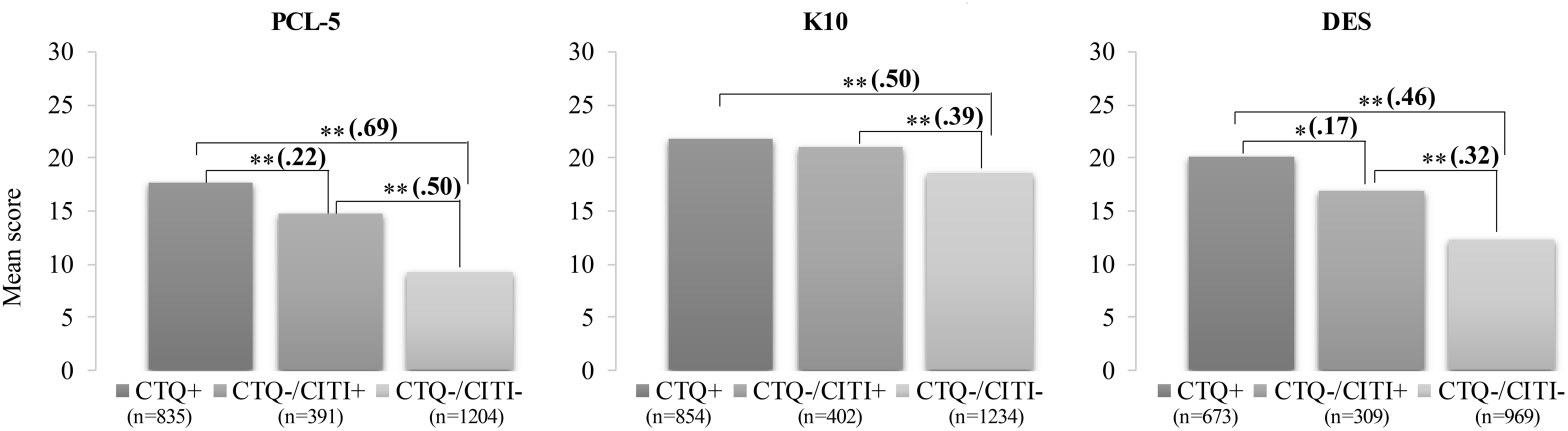
Group differences on the three outcomes according to the endorsement of the CTQ and/or the CITI criterion. CITI, Childhood Interpersonal Trauma Inventory; CTQ, Childhood Trauma Questionnaire; PCL-5, Post-traumatic Stress Disorder Checklist for DSM-5; K10, Kessler Psychological Distress Scale; DES, Dissociative Experiences Scale. Values in parentheses represent Cohen's d effect sizes (d = 0.20 = small effect; d = 0.50 = medium effect; d = 0.80 = large effect). Total N for PCL-5 = 2430; total N for K10 = 2490; total N for DES = 1951. *p < 0.05 **p < 0.001.

Participants were next split into three groups: (1) Positive at the CTQ, (2) Negative at the CTQ and Positive at the CITI, and (3) Negative at the CTQ and at the CITI. The ANOVAs revealed significant group differences for the three outcome variables [PTSD, F(2, 2427) = 130.32, p <.001, η2 = .097; anxiodepressive symptoms, F(2, 2487) = 69.04, p <.001, η2 = .053; and dissociation, F(2, 1948) = 46.53, p <.001, η2 = .046]. *Post-hoc* comparisons revealed significant differences between each group on all three outcomes with the exception of anxiodepressive symptoms for which no difference was observed between participants screening positive at the CTQ and those screening negative at the CTQ but positive at the CITI (**Figure 1**). Participants who did not reach the cut-off for abuse or neglect at the CTQ but who screened positive at the CITI reported significantly higher severity of PTSD, anxiodepressive, and dissociative symptoms than participants without trauma (negative at both questionnaires), suggesting that the CITI captures a subgroup of participants that is not detected by the CTQ but that cannot be considered as non-exposed to trauma.

## 4 Discussion

The primary aim of this study was to provide initial validation data on a new self-report screening instrument assessing simply and rapidly a broad range of potentially traumatic experiences during childhood. Our findings have shown that the CITI has adequate sensitivity and specificity compared to the gold-standard assessment and provides relevant information that allows us to identify trauma-exposed adults who would remain undetected using other instruments such as the CTQ.

First, the sensitivity and specificity values of the CITI to detect child maltreatment suggest that this instrument provides valid data that can be compared to those obtained using other well-established measures such as the CTQ. Encouragingly, the sensitivity and specificity of the CITI were higher than those reported by Schmidt and colleagues (63) for the ACE in their sample of 77 pregnant women with sensitivity ranging between 46.2% and 75.0% and specificity between 63.2% and 86.3%.

Importantly, the CITI showed very similar associations with post-traumatic stress, anxiodepressive, and dissociative symptoms as those observed when using the CTQ, and the latter only captured a very small part of variance in outcomes (between 0.3 and 0.7%) not already explained by the CITI. This supports the good convergent and incremental validity of the CITI and suggests that the CITI may be an interesting alternative to the CTQ, with the added benefit of capturing a greater diversity of potentially traumatic experiences.

The CITI was initially developed to detect participants who appeared to have experienced significant adversity not properly captured by the CTQ. As expected, we found that participants without trauma according to the CTQ but who screened positive at the CITI should not be considered as non-exposed to trauma. Indeed, the latter reported significantly higher scores on three outcomes, with small to medium effect sizes, than participants without trauma according to both instruments. This represented 16% (n = 407) of the participants in the current sample. Therefore, these people are not the exception or the few but represent a significant proportion of the population who may not receive the appropriate clinical services or may be misclassified in research projects or clinical trials.

Our finding offers additional support to Shenk and colleague’s (64) observation of a risk of contamination in trauma research, that is, when participants in the control group have been exposed to the same event as participants in the experimental condition. Indeed, our demonstration that participants screening positive at the CITI and negative at the CTQ are more similar to CTQ positive participants than to non-exposed participants according to both instruments would suggest that current research may underestimate the magnitude of the effect of trauma by minimizing between-group differences (65). By providing a more extensive evaluation of multiple types of potentially traumatic experiences, the CITI may also be an interesting measure for researchers interested in the cumulative or synergetic effect of trauma (66, 67). Indeed, previous studies have shown that some trauma would have few effects when occurring in isolation but may have many more dramatic impacts when paired with other traumatic experiences (67), or when added to an already challenging life trajectory (68). This supports the need for a thorough assessment of exposure to multiple forms of trauma and not solely the types that are subjectively considered to be the most severe.

The current study has limitations. First, the sample consists mainly of white, highly educated, non-single women with high household income whereas men were under-represented. Further studies should replicate the findings using a more diverse sample to ensure the generalizability of the results. Second, it would have been preferable to verify the sensitivity and specificity of the CITI using an interview as a standard measure. Although the CTQ is a widely accepted measure used in the scientific literature, it is not as reliable as an interview. Therefore, the sensitivity and specificity analyses performed on the CITI represent more of an agreement ratio with the best-established measure of maltreatment. Further confirmation of the construct validity of the instrument should consider using a semi-structured interview covering the same experiences as those assessed in the CITI. Third, the outcome variables all consisted of psychiatric symptoms. Future studies should include more diverse measures of functioning to assess the contributions of considering multiple types of potentially traumatic experiences extending beyond abuse and neglect.

## 5 Conclusion

Although several measures of interpersonal traumas are well established in the scientific literature, we found that a measure that could cover simply and rapidly a broad range of potentially traumatic experiences was lacking. We created the Childhood Interpersonal Trauma Inventory (CITI) for that specific purpose. The CITI showed adequate sensitivity and specificity as well as good convergent and incremental validity when compared to the Childhood Trauma Questionnaire (CTQ). Of utmost importance, the CITI identified a significant number of participants who were considered without trauma at the CTQ but who encountered numerous (≥4) potentially traumatic experiences resulting in significant psychiatric difficulties. Therefore, the CITI is a tool that can be used alone or in conjunction with other measures to identify people who require special clinical attention.

## Data availability statement

The raw data supporting the conclusions of this article will be made available by the authors, without undue reservation.

## Ethics statement

The studies involving humans were approved by Université du Québec à Trois-Rivières and CIUSSS Mauricie-et-du-Centre-du-Québec. The studies were conducted in accordance with the local legislation and institutional requirements. The participants provided their written informed consent to participate in this study.

## Author contributions

ML: Conceptualization, Data curation, Formal analysis, Methodology, Software, Validation, Visualization, Writing – original draft, Writing – review & editing. TM: Conceptualization, Methodology, Validation, Writing – review & editing. MR: Conceptualization, Formal analysis, Methodology, Validation, Writing – review & editing. RL: Validation, Writing – review & editing. JG-B: Data curation, Investigation, Project administration, Software, Validation, Writing – review & editing. NB: Conceptualization, Data curation, Funding acquisition, Methodology, Project administration, Resources, Supervision, Validation, Writing – review & editing.

## Footnotes

1 Lemieux R, Berthelot N. Inventaire des Traumatismes Interpersonnels vécus au cours de l’Enfance. Université du Québec à Trois-Rivières, QC. (Unpublished).

## References

[1] Afifi TO MacMillan HL Boyle M Taillieu T Cheung K Sareen J. Child abuse and mental disorders in Canada. Can Med Assoc J. (2014) 186:E324–32. doi: 10.1503/cmaj.131792 PMC4050024 24756625

[2] Garon-Bissonnette J Bolduc MG Lemieux R Berthelot N. Cumulative childhood trauma and complex psychiatric symptoms in pregnant women and expecting men. BMC Pregnancy Childbirth. (2022) 22:10. doi: 10.1186/s12884-021-04327-x 34983417 PMC8725451

[3] Gilbert R Widom CS Browne K Fergusson D Webb E Janson S. Burden and consequences of child maltreatment in high-income countries. Lancet. (2009) 373:68–81. doi: 10.1016/S0140-6736(08)61706-7 19056114

[4] Jaffee SR. Child maltreatment and risk for psychopathology in childhood and adulthood. Annu Rev Clin Psychol. (2017) 13:525–51. doi: 10.1146/annurev-clinpsy-032816-045005 28375720

[5] Archer G Pinto Pereira S Power C. Child maltreatment as a predictor of adult physical functioning in a prospective British birth cohort. BMJ Open. (2017) 7:e017900. doi: 10.1136/bmjopen-2017-017900 PMC5665268 29079607

[6] d'Huart D Hutsebaut J Seker S Schmid M Schmeck K Bürgin D. Personality functioning and the pathogenic effect of childhood maltreatment in a high-risk sample. Child Adolesc Psychiatry Ment Health. (2022) 16:95. doi: 10.1186/s13034-022-00527-1 36451183 PMC9710065

[7] Pfaltz MC Halligan SL Haim-Nachum S Sopp MR Åhs F Bachem R. Social functioning in individuals affected by childhood maltreatment: Establishing a research agenda to inform interventions. Psychother Psychosom. (2022) 91:238–51. doi: 10.1159/000523667 PMC9393832 35381589

[8] Savage LÉ Tarabulsy GM Pearson J Collin-Vézina D Gagné LM. Maternal history of childhood maltreatment and later parenting behavior: A meta-analysis. Dev Psychopathol. (2019) 31:9–21. doi: 10.1017/S0954579418001542 30757988

[9] Assink M Spruit A Schuts M Lindauer R van der Put CE Stams GJM. The intergenerational transmission of child maltreatment: A three-level meta-analysis. Child Abuse Negl. (2018) 84:131–45. doi: 10.1016/j.chiabu.2018.07.037 30086419

[10] van IJzendoorn MH Bakermans-Kranenburg MJ Coughlan B Reijman S. Annual Research Review: Umbrella synthesis of meta-analyses on child maltreatment antecedents and interventions: Differential susceptibility perspective on risk and resilience. J Child Psychol Psychiatry. (2020) 61:272–90. doi: 10.1111/jcpp.13147 PMC7065145 31667862

[11] Danese A Baldwin JR. Hidden Wounds? Inflammatory links between childhood trauma and psychopathology. Annu Rev Psychol. (2017) 68:517–44. doi: 10.1146/annurev-psych-010416-044208 27575032

[12] Hostinar CE Swartz JR Alen NV Guyer AE Hastings PD. The role of stress phenotypes in understanding childhood adversity as a transdiagnostic risk factor for psychopathology. J Psychopathol Clin Sci. (2023) 132:277–86. doi: 10.1037/abn0000619 PMC10153067 37126060

[13] Teicher MH Samson JA. Childhood maltreatment and psychopathology: A case for ecophenotypic variants as clinically and neurobiologically distinct subtypes. Am J Psychiatry. (2013) 170:1114–33. doi: 10.1176/appi.ajp.2013.12070957 PMC3928064 23982148

[14] Alvarez MJ Roura P Osés A Foguet Q Solà J Arrufat FX. Prevalence and clinical impact of childhood trauma in patients with severe mental disorders. J Nerv Ment Dis. (2011) 199:156–61. doi: 10.1097/NMD.0b013e31820c751c 21346485

[15] Larsson S Andreassen OA Aas M Røssberg JI Mork E Steen NE. High prevalence of childhood trauma in patients with schizophrenia spectrum and affective disorder. Compr Psychiatry. (2013) 54:123–7. doi: 10.1016/j.comppsych.2012.06.009 22901835

[16] Nanni V Uher R Danese A. Childhood maltreatment predicts unfavorable course of illness and treatment outcome in depression: A meta-analysis. Am J Psychiatry. (2012) 169:141–51. doi: 10.1176/appi.ajp.2011.11020335 22420036

[17] Nelson J Klumparendt A Doebler P Ehring T. Childhood maltreatment and characteristics of adult depression: Meta-analysis. Br J Psychiatry. (2017) 210:96–104. doi: 10.1192/bjp.bp.115.180752 27908895

[18] Lippard ETC Nemeroff CB. The devastating clinical consequences of child abuse and neglect: Increased disease vulnerability and poor treatment response in mood disorders. Am J Psychiatry. (2020) 177:20–36. doi: 10.1176/appi.ajp.2019.19010020 31537091 PMC6939135

[19] Teicher MH Gordon JB Nemeroff CB. Recognizing the importance of childhood maltreatment as a critical factor in psychiatric diagnoses, treatment, research, prevention, and education. Mol Psychiatry. (2022) 27:1331–8. doi: 10.1038/s41380-021-01367-9 PMC8567985 34737457

[20] Finkelhor D. Screening for adverse childhood experiences (ACEs): Cautions and suggestions. Child Abuse Negl. (2018) 85:174–9. doi: 10.1016/j.chiabu.2017.07.016 28784309

[21] McLennan JD MacMillan HL Afifi TO McTavish J Gonzalez A Waddell C. Routine ACEs screening is NOT recommended. Paediatr Child Health. (2019) 24:272–3. doi: 10.1093/pch/pxz042 PMC6587422 31241059

[22] Racine N Killam T Madigan S. Trauma-informed care as a universal precaution: Beyond the Adverse Childhood Experiences Questionnaire. JAMA Pediatr. (2020) 174:5–6. doi: 10.1001/jamapediatrics.2019.3866 31682717

[23] Berthelot N Lemieux R Maziade M. Shortfall of intervention research over correlational research in childhood maltreatment: An impasse to be overcome. JAMA Pediatr. (2019) 173:1009–10. doi: 10.1001/jamapediatrics.2019.1684 31524929

[24] Keeshin BR Monson E. Assessing and responding to the trauma of child maltreatment. Focus. (2022) 20:176–83. doi: 10.1176/appi.focus.20210033 PMC10153498 37153127

[25] McCrory EJ Gerin MI Viding E. Annual Research Review: Childhood maltreatment, latent vulnerability and the shift to preventative psychiatry - the contribution of functional brain imaging. J Child Psychol Psychiatry. (2017) 58:338–57. doi: 10.1111/jcpp.12713 PMC6849838 28295339

[26] Oral R Ramirez M Coohey C Nakada S Walz A Kuntz A. Adverse childhood experiences and trauma informed care: The future of health care. Pediatr Res. (2016) 79:227–33. doi: 10.1038/pr.2015.197 26460523

[27] Arseneault L. Annual Research Review: The persistent and pervasive impact of being bullied in childhood and adolescence: Implications for policy and practice. J Child Psychol Psychiatry. (2018) 59:405–21. doi: 10.1111/jcpp.12841 PMC6542665 29134659

[28] Idsoe T Vaillancourt T Dyregrov A Hagen KA Ogden T Nærde A. Bullying victimization and trauma. Front Psychiatry. (2021) 11:480353. doi: 10.3389/fpsyt.2020.480353 33519533 PMC7841334

[29] Nansel TR Overpeck M Pilla RS Ruan WJ Simons-Morton B Scheidt P. Bullying behaviors among US youth: Prevalence and association with psychosocial adjustment. JAMA. (2001) 285:2094–100. doi: 10.1001/jama.285.16.2094 PMC2435211 11311098

[30] Sigurdson JF Undheim AM Wallander JL Lydersen S Sund AM. The loqng-term effects of being bullied or a bully in adolescence on externalizing and internalizing mental health problems in adulthood. Child Adolesc Psychiatry Ment Health. (2015) 9:42. doi: 10.1186/s13034-015-0075-2 26300969 PMC4546259

[31] Wolke D Tippett N Dantchev S. Bullying in the family: Sibling bullying. Lancet Psychiatry. (2015) 2:917–29. doi: 10.1016/S2215-0366(15)00262-X 26462226

[32] Ossa FC Pietrowsky R Bering R Kaess M. Symptoms of posttraumatic stress disorder among targets of school bullying. Child Adolesc Psychiatry Ment Health. (2019) 13:43. doi: 10.1186/s13034-019-0304-1 31728159 PMC6842197

[33] Copeland WE Wolke D Angold A Costello EJ. Adult psychiatric outcomes of bullying and being bullied by peers in childhood and adolescence. JAMA Psychiatry. (2013) 70:419–26. doi: 10.1001/jamapsychiatry.2013.504 PMC3618584 23426798

[34] Macfie J Brumariu LE Lyons-Ruth K. Parent–child role-confusion: A critical review of an emerging concept. Dev Rev. (2015) 36:34–57. doi: 10.1016/j.dr.2015.01.002

[35] Van Loon LM Van de Ven MO Van Doesum KT Hosman CM Witteman CL. Parentification, stress, and problem behavior of adolescents who have a parent with mental health problems. Fam Process. (2017) 56:141–53. doi: 10.1111/famp.12165 26208046

[36] Doroudchi A Zarenezhad M Hosseininezhad H Malekpour A Ehsaei Z Kaboodkhani R. Psychological complications of the children exposed to domestic violence: A systematic review. Egypt J Forensic Sci. (2023) 13:26. doi: 10.1186/s41935-023-00343-4 37274510 PMC10213576

[37] Kuppens S Moore SC Gross V Lowthian E Siddaway AP. The enduring effects of parental alcohol, tobacco, and drug use on child well-being: A multilevel meta-analysis. Dev Psychopathol. (2020) 32:765–78. doi: 10.1017/S0954579419000749 PMC7525110 31274064

[38] Ranning A Madsen T Hawton K Nordentoft M Erlangsen A. Transgenerational concordance in parent-to-child transmission of suicidal behaviour: A retrospective, nationwide, register-based cohort study of 4 419 642 individuals in Denmark. Lancet Psychiatry. (2022) 9:363–74. doi: 10.1016/S2215-0366(22)00042-6 35354063

[39] Harman JJ Kruk E Hines DA. Parental alienating behaviors: An unacknowledged form of family violence. Psychol Bull. (2018) 144:1275–99. doi: 10.1037/bul0000175 30475019

[40] Verhaar S Matthewson ML Bentley C. The impact of parental alienating behaviours on the mental health of adults alienated in childhood. Children. (2022) 9:475. doi: 10.3390/children9040475 35455519 PMC9026878

[41] de Roo M Veenstra R Kretschmer T. Internalizing and externalizing correlates of parental overprotection as measured by the EMBU: A systematic review and meta-analysis. Soc Dev. (2022) 31:962–83. doi: 10.1111/sode.12590 PMC9790597 36588978

[42] Bernstein DP Stein JA Newcomb MD Walker E Pogge D Ahluvalia T. Development and validation of a brief screening version of the Childhood Trauma Questionnaire. Child Abuse Negl. (2003) 27:169–90. doi: 10.1016/s0145-2134(02)00541-0 12615092

[43] Felitti VJ Anda RF Nordenberg D Williamson DF Spitz AM Edwards V. Relationship of childhood abuse and household dysfunction to many of the leading causes of death in adults. The Adverse Childhood Experiences (ACE) Study. Am J Prev Med. (1998) 14:245–58. doi: 10.1016/s0749-3797(98)00017-8 9635069

[44] McLennan JD MacMillan HL Afifi TO. Questioning the use of adverse childhood experiences (ACEs) questionnaires. Child Abuse Negl. (2020) 101:104331. doi: 10.1016/j.chiabu.2019.104331 31887655

[45] Dobrow MJ Hagens V Chafe R Sullivan T Rabeneck L. Consolidated principles for screening based on a systematic review and consensus process. CMAJ. (2018) 190:E422–9. doi: 10.1503/cmaj.171154 PMC5893317 29632037

[46] Berthelot N Drouin-Maziade C Garon-Bissonnette J Lemieux R Sériès T Lacharité C. Evaluation of the acceptability of a prenatal program for women with histories of childhood trauma: The program STEP. Front Psychiatry. (2021) 12:772706. doi: 10.3389/fpsyt.2021.772706 34803778 PMC8600135

[47] Paquette D Laporte L Bigras M Zoccolillo M. Validation de la version française du CTQ et prévalence de l’histoire de maltraitance. Sante Ment Que. (2004) 29:201–20. doi: 10.7202/008831ar 15470573

[48] Walker EA Unutzer J Rutter C Gelfand A Saunders K VonKorff M. Costs of health care use by women HMO members with a history of childhood abuse and neglect. Arch Gen Psychiatry. (1999) 56:609–13. doi: 10.1001/archpsyc.56.7.609 10401506

[49] Georgieva S Tomas JM Navarro-Pérez JJ. Systematic review and critical appraisal of Childhood Trauma Questionnaire - Short Form (CTQ-SF). Child Abuse Negl. (2021) 120:105223. doi: 10.1016/j.chiabu.2021.105223 34352686

[50] Saini SM Hoffmann CR Pantelis C Everall IP Bousman CA. Systematic review and critical appraisal of child abuse measurement instruments. Psychiatry Res. (2019) 272:106–13. doi: 10.1016/j.psychres.2018.12.068 30580133

[51] Ashbaugh AR Houle-Johnson S Herbert C El-Hage W Brunet A. Psychometric validation of the English and French versions of the Posttraumatic Stress Disorder Checklist for DSM-5 (PCL-5). PloS One. (2016) 11:e0161645. doi: 10.1371/journal.pone.0161645 27723815 PMC5056703

[52] Wilkins KC Lang AJ Norman SB. Synthesis of the psychometric properties of the PTSD checklist (PCL) military, civilian, and specific versions. Depress Anxiety. (2011) 28:596–606. doi: 10.1002/da.20837 21681864 PMC3128669

[53] Bovin MJ Marx BP Weathers FW Gallagher MW Rodriguez P Schnurr PP. Psychometric properties of the PTSD Checklist for Diagnostic and Statistical Manual of Mental Disorders-Fifth Edition (PCL-5) in veterans. Psychol Assess. (2016) 28:1379–91. doi: 10.1037/pas0000254 26653052

[54] Camirand H Traoré I Baulne J. Enquête québécoise sur la santé de la population, 2014-2015. 2e éd. Québec, Canada: Institut de la Statistique du Québec (2016). Available at: https://statistique.quebec.ca/fr/fichier/enquete-quebecoise-sur-la-sante-de-la-population-2014-2015-pour-en-savoir-plus-sur-la-sante-des-quebecois-resultats-de-la-deuxieme-edition.pdf.

[55] Kessler RC Andrews G Colpe LJ Hiripi E Mroczek DK Normand SL. Short screening scales to monitor population prevalences and trends in non-specific psychological distress. Psychol Med. (2002) 32:959–76. doi: 10.1017/s0033291702006074 12214795

[56] Andrews G Slade T. Interpreting scores on the kessler psychological distress scale (K10). Aust N Z J Public Health. (2001) 25:494–7. doi: 10.1111/j.1467-842x.2001.tb00310.x 11824981

[57] Spies G Stein DJ Roos A Mostert J Seedat S Vythilingum B. Validity of the Kessler 10 (K-10) in detecting DSM-IV defined mood and anxiety disorders among pregnant women. Arch Womens Ment Health. (2009) 12:69–74. doi: 10.1007/s00737-009-0050-0 19238521

[58] Larøi F Billieux J Defeldre A-C Ceschi G van der Linden M. Factorial structure and psychometric properties of the French adaptation of the Dissociative Experiences Scale (DES) in non-clinical participants. Eur Rev Appl Psychol. (2013) 63:203–8. doi: 10.1016/j.erap.2013.04.004

[59] Bernstein EM Putnam FW. Development, reliability, and validity of a dissociation scale. J Nerv Ment Dis. (1986) 174:727–35. doi: 10.1097/00005053-198612000-00004 3783140

[60] Carlson EB Putnam FW Ross CA Torem M Coons P Dill DL. Validity of the Dissociative Experiences Scale in screening for multiple personality disorder: A multicenter study. Am J Psychiatry. (1993) 150:1030–6. doi: 10.1176/ajp.150.7.1030 8317572

[61] Campbell TL. Screening for Adverse Childhood Experiences (ACEs) in primary care: A cautionary note. JAMA. (2020) 323:2379–80. doi: 10.1001/jama.2020.4365 32463425

[62] Hughes K Bellis MA Hardcastle KA Sethi D Butchart A Mikton C. The effect of multiple adverse childhood experiences on health: A systematic review and meta-analysis. Lancet Public Health. (2017) 2:e356–66. doi: 10.1016/S2468-2667(17)30118-4 29253477

[63] Schmidt MR Narayan AJ Atzl VM Rivera LM Lieberman AF. Childhood maltreatment on the Adverse Childhood Experiences (ACEs) scale versus the Childhood Trauma Questionnaire (CTQ) in a perinatal sample. J Aggress Maltreat Trauma. (2020) 29:38–56. doi: 10.1080/10926771.2018.1524806

[64] Shenk CE Rausch JR Shores KA Allen EK Olson AE. Controlling contamination in child maltreatment research: Impact on effect size estimates for child behavior problems measured throughout childhood and adolescence. Dev Psychopathol. (2022) 34:1287–99. doi: 10.1017/S0954579420002242 PMC8440661 33719996

[65] Marfo P Okyere GA. The accuracy of effect-size estimates under normals and contaminated normals in meta-analysis. Heliyon. (2019) 5:e01838. doi: 10.1016/j.heliyon.2019.e01838 31211256 PMC6562325

[66] Briere J Kaltman S Green BL. Accumulated childhood trauma and symptom complexity. J Trauma Stress. (2008) 21:223–6. doi: 10.1002/jts.20317 18404627

[67] Briggs EC Amaya-Jackson L Putnam KT Putnam FW. All adverse childhood experiences are not equal: The contribution of synergy to adverse childhood experience scores. Am Psychol. (2021) 76:243–52. doi: 10.1037/amp0000768 33734792

[68] Mock SE Arai SM. Childhood trauma and chronic illness in adulthood: Mental health and socioeconomic status as explanatory factors and buffers. Front Psychol. (2011) 1:246. doi: 10.3389/fpsyg.2010.00246 21833299 PMC3153850

