# Supplementary material for: Beyond abuse and neglect: validation of the childhood interpersonal trauma inventory in a community sample of adults
Source: Front Psychiatry. 2024 Feb 29;15:1358475. doi: 10.3389/fpsyt.2024.1358475 (PMC10937553; doi:10.3389/fpsyt.2024.1358475)
Supplement: Supplementary file 1 [file DataSheet_1.docx]

**Supplementary Material: CITI items in English**

1. Experiencing bullying on a prolonged basis.

2. Being hit by a parent.

3. Being bullied more than once by a youth without anyone intervening.

4. Being afraid of a family member.

5. Living with a parent who was mentally ill.

6. Abandonment by a parent or prolonged interruption of contact.

7. Being threatened with abandonment or foster care.

8. Death of a parent.

9. Attempted suicide of a parent.

10. Attempted suicide of a close relative.

11. Incarceration of a relative.

12. Drug or alcohol abuse by a parent.

13. Had sexual contact with someone who was older by 2 years (if respondent was <14 years) or 5 years (if respondent was between 14-16 years).

14. Have had unwanted sexual contact with someone of the same age or younger.

15. Witnessed sexual intercourse or been exposed to pornography by an adult.

16. Being yelled at or openly belittled by a parent.

17. Being threatened with physical violence within the family.

18. Witnessing physical violence between parents.

19. Witnessing verbal abuse between parents.

20. Living with a parent who was very emotionally cold.

21. Living with a parent who could sulk several days in a row.

22. Feeling that one's presence in the family was unwanted.

23. Feeling that one's emotions were not important or not listened to.

24. Feeling that one's privacy was not respected.

25. One parent was overprotective or controlling.

26. Lacking food or having to wear dirty clothes.

27. Significant poverty situation.

28. Feeling as one that was not being taken care of.

29. Having to care for a parent because they were sick or unable to care for themselves.

30. Not receiving needed physical or psychological health care.

31. Being treated as a partner by one of the parents.

32. One parent was trying to turn you against the other parent.

33. Being punished excessively.

**Supplementary Material: CITI items that cover each type of childhood maltreatment**

When at least one item is endorsed, the type of childhood maltreatment it represents is considered endorsed.

**Sexual abuse**

13. Had sexual contact with someone who was older by 2 years (if respondent was <14 years) or 5 years (if respondent was between 14-16 years).

14. Have had unwanted sexual contact with someone of the same age or younger.

15. Witnessed sexual intercourse or been exposed to pornography by an adult.

**Physical abuse**

2. Being hit by a parent.

17. Being threatened with physical violence within the family.

33. Being punished excessively.

**Emotional abuse**

7. Being threatened with abandonment or foster care.

16. Being yelled at or openly belittled by a parent.

21. Living with a parent who could sulk several days in a row.

**Physical neglect**

12. Drug or alcohol abuse by a parent.

26. Lacking food or having to wear dirty clothes.

30. Not receiving needed physical or psychological health care.

**Emotional neglect**

20. Living with a parent who was very emotionally cold.

22. Feeling that one's presence in the family was unwanted.

23. Feeling that one's emotions were not important or not listened to.

28. Feeling as one that was not being taken care of.
